# The Clinical Education Partnership Initiative: an innovative approach to global health education

**DOI:** 10.1186/s12909-014-0246-5

**Published:** 2014-12-30

**Authors:** Aliza Monroe-Wise, Minnie Kibore, James Kiarie, Ruth Nduati, Joseph Mburu, Frederick Thurston Drake, William Bremner, King Holmes, Carey Farquhar

**Affiliations:** Department of Medicine, University of Washington, Seattle, USA; Department of Global Health, University of Washington, Seattle, USA; Department of Epidemiology, University of Washington, Seattle, USA; Department of Ob-Gyn, University of Nairobi, Nairobi, Kenya; Department of Pediatrics, University of Nairobi, Nairobi, Kenya; Naivasha District Hospital, Naivasha, Kenya; Department of Surgery, University of Washington, Seattle, USA

**Keywords:** International, Clinical rotation, Medical education, Residents, Kenya

## Abstract

**Background:**

Despite evidence that international clinical electives can be educationally and professionally beneficial to both visiting and in-country trainees, these opportunities remain challenging for American residents to participate in abroad. Additionally, even when logistically possible, they are often poorly structured. The Universities of Washington (UW) and Nairobi (UoN) have enjoyed a long-standing research collaboration, which recently expanded into the UoN Medical Education Partnership Initiative (MEPI). Based on MEPI in Kenya, the Clinical Education Partnership Initiative (CEPI) is a new educational exchange program between UoN and UW. CEPI allows UW residents to partner with Kenyan trainees in clinical care and teaching activities at Naivasha District Hospital (NDH), one of UoN’s MEPI training sites in Kenya.

**Methods:**

UW and UoN faculty collaborated to create a curriculum and structure for the program. A Chief Resident from the UW Department of Medicine coordinated the program at NDH. From August 2012 through April 2014, 32 UW participants from 5 medical specialties spent between 4 and 12 weeks working in NDH. In addition to clinical duties, all took part in formal and informal educational activities. Before and after their rotations, UW residents completed surveys evaluating clinical competencies and cross-cultural educational and research skills. Kenyan trainees also completed surveys after working with UW residents for three months.

**Results:**

UW trainees reported a significant increase in exposure to various tropical and other diseases, an increased sense of self-reliance, particularly in a resource-limited setting, and an improved understanding of how social and cultural factors can affect health. Kenyan trainees reported both an increase in clinical skills and confidence, and an appreciation for learning a different approach to patient care and professionalism.

**Conclusions:**

After participating in CEPI, both Kenyan and US trainees noted improvement in their clinical knowledge and skills and a broader understanding of what it means to be clinicians. Through structured partnerships between institutions, educational exchange that benefits both parties is possible.

**Electronic supplementary material:**

The online version of this article (doi:10.1186/s12909-014-0246-5) contains supplementary material, which is available to authorized users.

## Background

### International health electives

International health electives (IHEs) in some settings have been shown to increase United States (US) medical trainees’ understanding of public health, primary care, health disparities and barriers to care, in addition to raising National Board of Medical Examiners (NBME) scores and improving physical exam and history taking skills [1]. As such, both the demand for and the prevalence of these experiences are increasing among medical trainees [2]. Currently, 87% of US medical schools offer international clinical experiences to students, and it is estimated that over 25% of students participate in these programs [3,4]. Graduating medical students throughout all disciplines rank the availability of international experiences as among the most important factors in their choice of residency program [5]. However, due to the rigors of residency training and a number of other constraints, only 59% of residency programs offer international clinical rotations to residents, and as few as 10% of residents may be able to take advantage of these opportunities, when they exist [4].

Pitfalls for IHEs over the last several decades have been described. A common problem inherent to international clinical experiences is poorly defined academic goals and clinical competencies [3]. Another commonly cited issue, especially among medical students, is lack of structure and supervision, leading to concerns that one’s presence in the host institution may be detrimental to patient care [6]. Finally, concerns have been raised that short-term international clinical experiences might benefit the sending institution and its trainees more than that of the host institution, and that benefits for the host institution can be difficult to measure [7].

Medical training in most parts of sub-Saharan Africa includes 6 years of undergraduate medical school and one year of internship, in which newly graduated clinicians (medical officer interns) are placed in government hospitals to practice. Inadequate numbers of medical training facilities and faculty positions have been identified as challenges facing medical education in sub-Saharan Africa [8], leading to trainees with limited access to senior clinicians for both teaching and role modeling. Formal training programs involving regularly scheduled, structured group didactics for doctors recruited to work in rural Africa have been shown to increase “professional socialization,” or the “learning of attitudes, norms, self images, values, beliefs and behavioral patterns associated with professional practice,” in addition to clinical knowledge and skills [9,10]. This enhanced sense of connection was thought to be related to the mode of training rather than the content, and led to long-term professional relationships and increased job satisfaction [9]. Despite these advantages, there are numerous barriers in place for structured continuing medical education in most healthcare facilities in sub-Saharan Africa [11].

Given the relative lack of international clinical opportunities for US residents, the limitations on medical education in much of sub-Saharan Africa and the logistical and ethical considerations that have been described pertaining to IHEs for medical students, the Universities of Washington and Nairobi have recently initiated a new clinical training program for UW and UoN residents in Naivasha, Kenya. This one-month rotation is based on a long-term, structured educational exchange partnership, and aims to provide trainees with supervised, hands-on experience based on a predefined curriculum covering four main competencies of healthcare delivery in resource-poor settings.

### Partnership for innovative medical education in Kenya

Researchers at the University of Washington (UW) have collaborated with researchers from the University of Nairobi (UoN) on various projects for over 30 years [12]. This longstanding collaboration recently culminated in a Medical Education Partnership Initiative (MEPI) grant from the National Institutes of Health and PEPFAR. The new initiative, called Partnership for Innovative Medical Education in Kenya, or PRIME-K, is designed to strengthen medical education, improve retention of clinicians in rural parts of Kenya, and foster research capacity in Kenya. Modeled on the Washington, Wyoming, Alaska, Montana and Idaho (WWAMI) Regional Medical Education Program, which provides medical education for all 5 states and extends medical training to very rural and remote areas [13,14], a new decentralized training system has been created for UoN medical students [15]. Since 2010, 238 medical students from UoN have completed rotations in 14 hospitals throughout the country, on both the district and provincial levels. Through the foundation of PRIME-K, the UW-UoN partnership has now launched a new initiative called the Clinical Education Partnership Initiative, which focuses on cross-cultural clinical education experiences in Kenya.

## Methods

### Setting

Naivasha District Hospital (NDH) was chosen as a site for the Clinical Education Partnership Initiative (CEPI), due to its proximity to Nairobi and its success with the establishment of a PRIME-K rotation for UoN medical students. NDH is a Level 4 Ministry of Health facility that serves both the urban and rural populations of Naivasha, a catchment area of over 350,000 people [16]. In total, 7 consultants (attending physicians who are permanent employees) work at the hospital, including two surgeons, one otolaryngologist, one internist, one obstetrician-gynecologist, one radiologist, and one pediatrician. The facility currently has just over 200 beds, many of which are often occupied by two patients. There are two operating rooms, an urgent care center, an HIV clinic, a TB clinic, and a rotating consultant’s clinic where the specialists see their continuity patients. Faculty from both University of Washington and University of Nairobi met with the Ministry of Health and the Medical Superintendent of the hospital, who approved the partnership.

### Curriculum

Prior to program implementation, UW and UoN faculty collaborated to develop a curriculum outlining the goals and competencies that UW residents would be expected to achieve during rotations. Drawing on both faculty experience and resources for global health education from both institutions, four main objectives were defined: Clinical Skills & Knowledge, Education & Mentorship, Community Health Work, and Research & Partnerships. Within each area, specific educational goals for residents were established and the activities of the rotation were designed to achieve the goals.

### Structure

**Figure 1:**
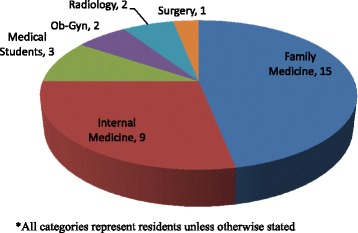
Description of UW participants*.

A Global Health Chief Resident from the UW Department of Medicine was appointed and moved to Naivasha to establish the program in August 2012, and the first residents arrived in September 2012. The Chief Resident organized all logistics of the program for the participants, including transportation, housing, educational activities, and clinical work. The rotation was open to any resident in any department of the University of Washington, and select medical students identified by the medical school’s global health track. Twenty-nine residents from 5 departments and three medical students have completed rotations between September 2012 and April 2014 (see Figure 1). All residents were in their second or third year of residency (out of 3 years for internal and family medicine, 4 years for Ob-Gyn, and 6 years for surgery), and the students were in the end of their fourth year. Each resident attended an orientation in Seattle prior to departure and another one in Naivasha on arrival. Housing and within-country transportation were provided for the UW trainees. The majority of residents spent 4 weeks in Naivasha, and participated in a number of clinical activities including hospital ward rounds, consultant’s outpatient clinic, emergency/urgent care, operating theatre, and dispensary visits. Additionally, UW trainees presented at least two educational lectures during their rotation and spent an average of 2–3 days in rural communities engaged in public health outreach activities. UW trainees worked side by side with their Kenyan colleagues in all settings, including educational conferences. Kenyan trainees and clinical staff participating in CEPI included medical officer interns (medical doctors who have completed a 6-year combined undergraduate & medical school degree), medical officers (medical doctors who have completed medical school and intern year but have not undergone specialty training in residency), clinical officers interns (midlevel providers who have completed 4-year clinical officer training), and medical students.

### Monitoring and evaluation

Faculty members from UW and UoN collaborated to create a monitoring and evaluation structure for both UW and NDH trainees prior to the start of the program. Because data were collected from program participants for quality control and program improvement purposes, data collection was not considered to be research by both the UW and UoN ethics committees, and ethical approval was not applicable. UW residents completed before- and after-rotation surveys (Additional files 1 and 2) that were designed to measure success in achieving the curricular goals of the program, which are outlined above. These were done anonymously; however, while the surveys did not include specific identifying information such as name, some of the questions may have provided information that would allow identification in certain circumstances. Additionally, UW residents were asked to complete a fully anonymous online evaluation of the rotation after their return to Seattle. Kenyan trainees completed anonymous surveys every 3 months describing their involvement with UW residents during each time period (Additional file 3). A group of Kenyan trainees also participated in one semi-structured focus group discussion. Surveys for UW and UoN trainees did not differ based on level of training. Survey scores were compiled in Microsoft Excel, and paired t-tests were used to determine significance of change.

## Results & discussion

### Naivasha district hospital trainees

Surveys were completed by NDH trainees four times, in November 2012, May and September 2013, and March 2014. A total of 50 surveys were completed. Of these, 26 were completed by clinical officer interns (COIs), 11 were completed by medical officer interns (MOIs), 10 were medical officers (MOs), two were students, and one was a clinical officer (CO). Trainees from NDH reported learning from UW residents most frequently during ward rounds, teaching conferences, and informal educational settings. Of all educational settings, they ranked surgical theatre and teaching conferences to be most helpful. 100% of NDH trainees reported feeling more confident about medical knowledge and more confident taking care of patients as a result of their interaction with UW residents.

Trainees at NDH most frequently cited improvements in diagnosis and management of patients when asked how their interactions with UW residents benefitted them. They specified that they learned “more etiologies to conditions,” how to “arrive at a particular diagnosis” using the “correct investigations,” and “coming up with differential [diagnoses]”. Others mentioned getting help with physical examination and “proper management” of patients from the UW residents. Commonly mentioned ways that UW residents helped with medical knowledge were updating, reinforcing, or broadening knowledge gained in medical school. Specific skills that were frequently mentioned were wound care, physical examination, problem-solving and procedures.

Many NDH trainees stated they appreciated getting a “different approach” to patient care from UW residents. This was mentioned both with respect to clinical decision making and to a broader sense of one’s approach to doctoring. One trainee mentioned that the most useful element of the partnership was “getting to know the alternative way of approaching the usual conditions, and not sticking to the one way of approach,” highlighting the opportunity to broaden one’s methods for clinical decision making. Others mentioned “different approach to get the diagnosis,” and “stimulating my thought process”.

Additionally, trainees alluded to learning a different approach to the art of doctoring, in both the patient-doctor relationship and professional relationships between clinicians. Teamwork and communication were stressed by several trainees, who stated that they appreciated learning “work distribution” and “importance of working as a team”. One trainee stated that UW residents were helpful in encouraging the trainee to “ask when I am not sure without fear”. With regard to the patient-doctor relationship, different Kenyan trainees mentioned that “talking to patients” and “how to give a patient maximum concern,” were examples of skills learned from this alternative approach, as was demonstrating “ideals of patient care,” and “holistic care”. One trainee stated that the residents were “a source of motivation. Because they don’t give up… they did everything they could do”. Another trainee simply stated, “I learnt the art of compassion”.

Overall, Kenyan trainees reported feeling supported by and more confident as a result of the program. “I can now make confident decisions when left unsupervised due to the acquired knowledge,” one mentioned. “I found it very interactive, if we teach those people what we know, and they teach us what we don’t know, there is going to be a working relationship”.

### University of Washington trainees

Of the 32 UW trainees who participated, 30 (94%) completed before and after surveys, making a total of 60 surveys; however, 5 “before” surveys were lost during a robbery, leaving 55 for analysis, including 25 complete before-and-after pairs. During their 4-week rotations, visiting trainees reported seeing significantly more cases of advanced HIV, tuberculosis, malaria, pediatric HIV, severe malnutrition, neonatal sepsis, and neonatal asphyxiation cases than they ever had previously during their medical training. On average, after their rotation U.S. trainees reported feeling more confident diagnosing disease based on history and physical examination, managing patients in a setting with limited resources, working in very different hospital systems, and using clinical guidelines for management of patients in resource limited settings (Table 1). U.S. trainees also reported engaging in certain educational activities significantly more often during the rotation than they had in their previous training (Table 2). These included teaching people of different cultural and linguistic backgrounds, working with colleagues from different cultural and linguistic backgrounds, and researching educational topics with few resources. Finally, U.S. trainees reported a significantly greater understanding of the complex role that social determinants can play in health, with significant improvements in understanding how poverty affects health and how economic, social and cultural barriers can affect health care seeking (Table 3).

**Table 1:** **UW Before- and after- rotation clinical competency scores**

Involvement in care of patients with:*	**Average score before rotation**	**Average score during rotation**	**Average difference in before/after**	**p-value§**
Advanced HIV	2.36	2.92	0.56	0.05
Tuberculosis	2.24	3.08	0.84	<0.01
Malaria	1.48	2.44	0.96	<0.01
Pediatric HIV	1.23	2.08	0.85	<0.01
Severe malnutrition	2.00	2.64	0.64	0.03
Neonatal sepsis	2.42	3.25	0.83	0.03
Neonatal asphyxia	1.42	2.92	1.50	<0.01
Severe diarrhea	1.73	2.36	0.63	0.01
Comfort with the following clinical skills:**				
Diagnosing disease based on history, physical, and vital signs	3.16	3.56	0.40	0.08
Managing patients with limited resources	2.60	3.56	0.96	<0.01
Working effectively in hospital systems different from in the USA	2.58	3.46	0.88	<0.01
Use of clinical guidelines for management	2.58	3.75	1.17	<0.01

*Scores based on a 5-point scale: 1 = 0 patients seen, 2 = 1-5 patients seen, 3 = 6-20 patients seen, 4 = 21-50 patients seen, 5 = Greater than 50 patients seen.

**Scores based on a 5-point scale: 1 = Not at all, 2 = A little bit, 3 = Somewhat, 4 = Reasonably, 5 = Very.

§P-values calculated from paired t-tests.

**Table 2:** UW Before- and after- rotation educational experience scores*

**Number of times engaged in the following activities:**	**Average score before rotation**	**Average score during rotation**	**Average difference in before/after**	**P-value§**
Teaching people of different cultural or linguistic backgrounds from your own	2.24	3.68	1.44	<0.01
Researching educational topics with few available resources	1.96	3.00	1.04	<0.01
Working with colleagues from different cultural backgrounds from your own	3.08	4.28	1.20	<0.01

*Scores based on a 5-point scale: 1 = Never, 2 = 1-5 Times, 3 = 6-20 Times, 4 = 21-50 Times, 5= >50 Times.

§P-values calculated from paired t-tests.

**Table 3:** UW Before- and after- rotation community health work scores*

**Level of understanding of:**	**Average score before rotation**	**Average score during rotation**	**Average difference in before/after**	**P-value§**
How extreme poverty affects health	3.13	4.13	1.00	<0.01
Economic barriers to health care seeking	3.33	4.21	0.88	<0.01
Social and cultural barriers to health care seeking	3.33	3.96	0.63	0.01

*Scores based on a 5-point scale: 1 = Not at all, 2 = A little bit, 3 = Somewhat, 4 = Reasonably, 5 = Very.

§P-values calculated from paired t-tests.

When asked what the most educational aspects of the rotation were, most U.S. trainees mentioned patient management with limited resources. “The experience of working in a low resource healthcare [setting] was the most valuable. The decisions I had to make with limited information or imaging taught me skills that I would not have obtained in my residency.” Other commonly cited aspects were the exposure to rare diagnoses such as advanced HIV and TB, and the opportunity to teach and motivate others at the hospital.

Twenty-nine (91%) out of 32 U.S. trainees reported experiencing a change in the way they viewed themselves as clinicians as a result of their rotation in Naivasha. The majority of these cited increased confidence and self-reliance as a clinician, especially when dealing with resource limitations. Other commonly expressed feelings were gratitude for the education, training and resources available in the U.S. and feeling more committed to careers in global health. Interestingly, while the majority of internal and family medicine residents felt more confidence, those in procedure-based residencies such as surgery and obstetrics-gynecology tended to feel more humbled by the experience. A surgery resident reported “I’m not more confident having had this experience that so stretched my comfort limits and my abilities. I’m actually more humble, with a new appreciation and hunger for the skills, wisdom, and judgment I have yet to learn as I continue my surgical training,” while an ob-gyn resident stated “I feel that medical education is a great gift that I have received and this rotation [made me want] to share this with others”.

“Challenging” and “rewarding” were the most frequently cited descriptions for the rotation overall, and these were usually mentioned together, implying that the challenges themselves provided the greatest rewards. The educational value was also highlighted, with trainees describing growth in clinical knowledge, understanding about disparities, and learning about the culture. One trainee described the rotation as “The single most unique, challenging and valuable rotation in my residency”.

## Conclusions

CEPI is an innovative new program designed to foster educational exchange between medical trainees from two vastly different academic backgrounds: University of Washington and University of Nairobi. The partnership between these two universities is founded in several decades of research collaboration, lending stability and long-term investment to the program. Additionally, educational supervision and support provided by the CEPI Chief Resident allows trainees to focus on clinical education, rather than logistics of living and practicing in a foreign setting for a short time. The Chief Resident also provides structure to the training program for both UW and Kenyan trainees, acts as a liaison between the two universities, and reduces the time that Kenyan consultants and administrators have to dedicate to the logistics of the program to essentially zero.

In its first year and a half, 32 UW trainees and up to 50 Kenyan trainees have benefitted from this exchange program. All participants from both countries reported having learned a great deal from the experience. There were several clinical and educational competencies that UW residents were able to practice more frequently in Kenya than in the USA. Many UW residents reported increased confidence. Other residents, particularly those from procedure-based specialties, described a re-kindled appreciation for the rigors of and supervision provided by specialty training back home.

The prevalent sense among Kenyan trainees that interactions with UW residents helped them by teaching “a different approach” is a critical finding. This “different approach,” whether referring to a clinician’s thought process, relationship with the patient, or relationship with a colleague, is as important for trainees in rural areas to learn as clinical knowledge. During these critical years of training, clinicians will develop habits that will likely inform their practice for years to come. Working with senior clinicians from different backgrounds can provide models for various professional behaviors, but this can be difficult to achieve in remote, resource-poor settings. By bringing UW residents to NDH, CEPI effectively provides a variety of professional role models to rural Kenyan trainees.

This study had several significant limitations. First, all results are self-reported measures of learning, and not patient care outcomes, which may be more pertinent and less vulnerable to various types of bias. Second, a significant amount of data was lost during a robbery, reducing the number of analyzable results. We limited our evaluation process to participants in the program and did not include perspectives from the hospital staff, who may also be affected by CEPI activities. Third, we did not evaluate any “control” group with which to compare our results, and therefore cannot definitively attribute any changes observed to our program. Finally, changes over time were not measured, so it is impossible to predict whether the effects may be limited.

As CEPI continues to bring UW residents to Naivasha, there are several further directions the program may take that might enhance and broaden the experience for both UW and Kenyan trainees. Although we have reported on only clinical work and education here, the curriculum encompasses two additional components of the program, and these should be evaluated in future years when more established. Both community health work and research are program elements that will help to round out resident experiences in future years of CEPI. Tele-learning through video broadcasting of teaching conferences would be a great addition to the existing teaching structure, and would add to the sense of partner institutions joined in learning. Residents working toward their Masters of Medicine degrees (MMed, equivalent to residency training in the US) at UoN will be joining UW residents for rotations at NDH in the future, increasing the sense of partnership among individual trainees at the same stage of training and broadening the opportunities for collaboration and research endeavors. Additionally, opportunities for NDH trainees to travel to Seattle to experience clinical rotations there would provide a true exchange, and much greater depth to the overall relationship between the two institutions. Through CEPI we have found a long-term, sustainable partnership for educational exchange to be beneficial to all parties involved; with the years to come, this promises to become a multi-faceted, profound educational experience that may change the career trajectories of clinicians in Kenya and Seattle alike.

## Additional files

Additional file 1:
**Pre-Naivasha Rotation Questionnaire.**
Additional file 2:
**Post-Naivasha Rotation Questionnaire.**
Additional file 3:
**CEPI Naivasha District Hospital Clinical Instruction Assessment.**
