# Supplementary material for: The Clinical Education Partnership Initiative: an innovative approach to global health education
Source: BMC Med Educ. 2014 Dec 30;14:1043. doi: 10.1186/s12909-014-0246-5 (PMC4335420; doi:10.1186/s12909-014-0246-5)
Supplement: Additional file 1: — Pre-Naivasha Rotation Questionnaire. [file 12909_2014_246_MOESM1_ESM.docx]

**Pre-Naivasha Rotation Questionnaire**

Section 1: Demographics

What is your current position? Student Resident Fellow Attending

What year are you in your current position? 1 2 3 4 5 >5

What is your specialty?

Medicine

Family Medicine

Surgery

Ob-Gyn

Radiology

Other (list)

How much time have you previously spent in developing countries? _______ months

Have you ever spent time doing clinical work in a developing country? Yes No

If so, for how many months? ______

Have you ever spent time doing health-related research in a developing country? Yes No

If so, for how many months? ______

Section 2: Clinical Competencies

During your medical training, how many times have you been involved in the care of patients with the following problems?

|  | 0 | 1-5 | 6-20 | 21-50 | >50 |
| --- | --- | --- | --- | --- | --- |
| Complications from advanced HIV |  |  |  |  |  |
| TB |  |  |  |  |  |
| Malaria |  |  |  |  |  |
| Parasitic infections |  |  |  |  |  |
| Vaccine-preventable diseases such as measles |  |  |  |  |  |
| Pediatric HIV |  |  |  |  |  |
| Severe malnutrition |  |  |  |  |  |
| Preeclampsia or eclampsia |  |  |  |  |  |
| Postpartum hemorrhage |  |  |  |  |  |
| Neonatal sepsis |  |  |  |  |  |
| Neonatal asphyxia |  |  |  |  |  |
| Severe diarrhea presenting with shock or severe dehydration |  |  |  |  |  |
| Bacterial meningitis |  |  |  |  |  |

How comfortable do you currently feel with the following clinical skills?

|  | Not at all | A little bit | Somewhat | Reasonably | Very |
| --- | --- | --- | --- | --- | --- |
| Diagnosing diseases based on history, physical exam and vital signs alone |  |  |  |  |  |
| Managing patients in a setting with limited resources |  |  |  |  |  |
| Working with interpreters |  |  |  |  |  |
| Managing patients of very different cultural backgrounds from your own |  |  |  |  |  |
| Working effectively in hospital systems very different from those in the US |  |  |  |  |  |
| Use of clinical guidelines for management of patients in resource limited settings |  |  |  |  |  |

Section 3: Education and Mentorship

How many times have you engaged in the following activities during your medical training?

|  | 0 | 1-5 | 6-20 | 21-50 | >50 |
| --- | --- | --- | --- | --- | --- |
| Teaching groups of different types of clinicians such as nurses, technicians or dietitians |  |  |  |  |  |
| Teaching people of different cultural or linguistic backgrounds from your own |  |  |  |  |  |
| Researching educational topics with few available resources |  |  |  |  |  |
| Working with colleagues of different cultural backgrounds from your own |  |  |  |  |  |

Section 4: Community Health Work

How well do you think you understand the following?

|  | Not at all | A little bit | Somewhat | Reasonably | Very |
| --- | --- | --- | --- | --- | --- |
| How extreme poverty affects health |  |  |  |  |  |
| Economic barriers to health care seeking |  |  |  |  |  |
| Social and cultural barriers to health care seeking |  |  |  |  |  |

How many times have you engaged in the following activities during your formal medical training?

|  | 0 | 1-5 | 6-20 | 21-50 | >50 |
| --- | --- | --- | --- | --- | --- |
| Visiting the communities of your patients |  |  |  |  |  |
| Visiting the homes of your patients |  |  |  |  |  |
| Visiting the work places of your patients |  |  |  |  |  |

Section 5: Research and Partnerships

How many times have you engaged in the following activities?

|  | 0 | 1-5 | 6-20 | 21-50 | >50 |
| --- | --- | --- | --- | --- | --- |
| Collaborating on research projects with colleagues from a different country |  |  |  |  |  |
| Collaborating on patient care with colleagues from a different country |  |  |  |  |  |
| Designing or implementing research projects in different countries |  |  |  |  |  |
