# Supplementary material for: The Clinical Education Partnership Initiative: an innovative approach to global health education
Source: BMC Med Educ. 2014 Dec 30;14:1043. doi: 10.1186/s12909-014-0246-5 (PMC4335420; doi:10.1186/s12909-014-0246-5)
Supplement: Additional file 2: — Post-Naivasha Rotation Questionnaire. [file 12909_2014_246_MOESM2_ESM.docx]

**Post-Naivasha Rotation Questionnaire**

Section 1: Demographics

How long did you spend in Naivasha? _____ weeks

Section 2: Clinical Competencies

During your rotation in Naivasha, how many times have you been involved in the care of patients with the following problems?

|  | 0 | 1-5 | 6-20 | 21-50 | >50 |
| --- | --- | --- | --- | --- | --- |
| Complications from advanced HIV |  |  |  |  |  |
| TB |  |  |  |  |  |
| Malaria |  |  |  |  |  |
| Parasitic infections |  |  |  |  |  |
| Vaccine-preventable diseases such as measles |  |  |  |  |  |
| Pediatric HIV |  |  |  |  |  |
| Severe malnutrition |  |  |  |  |  |
| Preeclampsia or eclampsia |  |  |  |  |  |
| Postpartum hemorrhage |  |  |  |  |  |
| Neonatal sepsis |  |  |  |  |  |
| Neonatal asphyxia |  |  |  |  |  |
| Severe diarrhea presenting with shock or severe dehydration |  |  |  |  |  |
| Bacterial meningitis |  |  |  |  |  |

How comfortable do you currently feel with the following clinical skills?

|  | Not at all | A little bit | Somewhat | Reasonably | Very |
| --- | --- | --- | --- | --- | --- |
| Diagnosing diseases based on history, physical exam and vital signs alone |  |  |  |  |  |
| Managing patients in a setting with limited resources |  |  |  |  |  |
| Working with interpreters |  |  |  |  |  |
| Managing patients from very different cultural backgrounds from your own |  |  |  |  |  |
| Working effectively in hospital systems very different from those in the US |  |  |  |  |  |
| Use of clinical guidelines for management of patients in resource limited settings |  |  |  |  |  |

Section 3: Education and Mentorship

How many times have you engaged in the following activities during your rotation in Naivasha?

|  | 0 | 1-5 | 6-20 | 21-50 | >50 |
| --- | --- | --- | --- | --- | --- |
| Teaching groups of different types of clinicians such as nurses, technicians or dietitians |  |  |  |  |  |
| Teaching people of different cultural or linguistic backgrounds from your own |  |  |  |  |  |
| Researching educational topics with few available resources |  |  |  |  |  |
| Working with colleagues from different cultural backgrounds from your own |  |  |  |  |  |

Section 4: Community Health Work

How well do you think you currently understand the following?

|  | Not at all | A little bit | Somewhat | Reasonably | Very |
| --- | --- | --- | --- | --- | --- |
| How extreme poverty affects health |  |  |  |  |  |
| Economic barriers to health care seeking |  |  |  |  |  |
| Social and cultural barriers to health care seeking |  |  |  |  |  |

How many times have you engaged in the following activities during your rotation in Naivasha?

|  | 0 | 1-5 | 6-20 | 21-50 | >50 |
| --- | --- | --- | --- | --- | --- |
| Visiting the communities of your patients |  |  |  |  |  |
| Visiting the homes of your patients |  |  |  |  |  |
| Visiting the work places of your patients |  |  |  |  |  |

Section 5: Research and Partnerships

How many times have you engaged in the following activities during your rotation in Naivasha?

|  | 0 | 1-5 | 6-20 | 21-50 | >50 |
| --- | --- | --- | --- | --- | --- |
| Collaborating on research projects with colleagues from a different country |  |  |  |  |  |
| Collaborating on patient care with colleagues from a different country |  |  |  |  |  |
| Designing or implementing research projects in different countries |  |  |  |  |  |

Section 6: Rotation Feedback

What were the most educational aspects of your rotation?

Has this rotation changed the way you view yourself as a clinician? Yes No

If so, can you describe how?

Has this rotation changed your desire to practice medicine in other countries? Yes No

If so, can you describe how?

How would you describe this rotation to a colleague?
