# Supplementary material for: The Clinical Education Partnership Initiative: an innovative approach to global health education
Source: BMC Med Educ. 2014 Dec 30;14:1043. doi: 10.1186/s12909-014-0246-5 (PMC4335420; doi:10.1186/s12909-014-0246-5)
Supplement: Additional file 3: — CEPI Naivasha District Hospital Clinical Instruction Assessment. [file 12909_2014_246_MOESM3_ESM.docx]

**CEPI Naivasha District Hospital Clinical Instruction Assessment**

What is your current position?

Student MO Intern MO CO Intern CO

What department have you been assigned to during the past month?

Medicine Surgery Ob-Gyn Paediatrics Other (list)

How often this month did you participate in the following educational activities **with University of Washington (UW) residents**? Please approximate.

|  | Nearly Every Day | Several Times per Week | Several Times per Month | Never |
| --- | --- | --- | --- | --- |
| Ward Rounds |  |  |  |  |
| Surgical Theatre |  |  |  |  |
| Consultant’s Clinic |  |  |  |  |
| Tuesday Afternoon Conference |  |  |  |  |
| Thursday Morning Report |  |  |  |  |
| Other Informal Educational Settings |  |  |  |  |

How helpful did you feel your UW colleagues were in the following educational settings?

|  | Not Helpful | Somewhat Helpful | Very Helpful | Extremely Helpful |
| --- | --- | --- | --- | --- |
| Ward Rounds |  |  |  |  |
| Surgical Theatre |  |  |  |  |
| Consultant’s Clinic |  |  |  |  |
| Tuesday Afternoon Conference |  |  |  |  |
| Thursday Morning Report |  |  |  |  |
| Other Informal Educational Settings |  |  |  |  |

Do you feel more confident about your medical knowledge now as a result of your interaction with a UW resident?

Yes No

If so, can you describe how the UW residents helped you with medical knowledge?

Do you feel more confident taking care of patients now as a result of your interaction with a UW resident?

Yes No

If so, can you describe how the UW residents helped you with patient care?

**Thank you for your feedback!**
